# Emergency department length of stay for patients requiring mechanical ventilation: a prospective observational study

**DOI:** 10.1186/1757-7241-20-30

**Published:** 2012-04-11

**Authors:** Louise Rose, Sara Gray, Karen Burns, Clare Atzema, Alex Kiss, Andrew Worster, Damon C Scales, Gordon Rubenfeld, Jacques Lee

**Affiliations:** 1 Lawrence S. Bloomberg Faculty of Nursing, 155 College St, Rm 276, Toronto, ON, M5T IP8, Canada; 2 Departments of Emergency Medicine and Critical Care, St Michaels Hospital, 30 Bond St, Toronto, ON, M5B 1W8, Canada; 3 Department of Critical Care, St Michaels Hospital, 30 Bond St., Toronto, ON, M5B 1W8, Canada; 4 Department of Trauma, Emergency and Critical Care, Sunnybrook Health Sciences Centre, 2075 Bayview Ave., Toronto, ON, M4N 3M5, Canada; 5 Department of Research Design and Biostatistics, Sunnybrook Health Sciences Centre, 2075 Bayview Ave, Toronto, ON, M4N 3M5, Canada; 6 Department of Emergency Medicine, Hamilton Health Sciences & McMaster University, 1200 Main St, West Hamilton, L8N 3Z5, Canada

**Keywords:** Mechanical ventilation, Emergency department, Non-invasive ventilation, critical illness, Acute respiratory failure

## Abstract

**Background:**

Recommendations for acceptable emergency department (ED) length of stay (LOS) vary internationally with ≤ 8 h generally considered acceptable. Protracted ED LOS may place critically ill patients requiring mechanical ventilation at increased risk of adverse events as most EDs are not resourced for longitudinal delivery of critical care. Our objective was to quantify the ED LOS for mechanically ventilated patients (invasive and/or non-invasive ventilation [NIV]) and to explore patient and system level predictors of prolonged ED LOS. Additionally, we aimed to describe delivery and monitoring of ventilation in the ED.

**Methods:**

Prospective observational study of ED LOS for all patients receiving mechanical ventilation at four metropolitan EDs in Toronto, Canada over two six-month periods in 2009 and 2010.

**Results:**

We identified 618 mechanically ventilated patients which represented 0.5% (95% CI 0.4%–0.5%) of all ED visits. Of these, 484 (78.3%) received invasive ventilation, 118 (19.1%) received NIV; 16 received both during the ED stay. Median Kaplan-Meier estimated duration of ED stay for all patients was 6.4 h (IQR 2.8–14.6). Patients with trauma diagnoses had a shorter median (IQR) LOS, 2.5 h (1.3–5.1), compared to ventilated patients with non-trauma diagnoses, 8.5 h (3.3–14.0) (*p* <0.001). Patients requiring NIV had a longer ED stay (16.6 h, 8.2–27.9) compared to those receiving invasive ventilation exclusively (4.6 h, 2.2–11.1) and patients receiving both (15.4 h, 6.4–32.6) (*p* <0.001). Longer ED LOS was associated with ED site and lower priority triage scores. Shorter ED LOS was associated with intubation at another ED prior to transfer.

**Conclusions:**

While patients requiring mechanical ventilation represent a small proportion of overall ED visits these critically ill patients frequently experienced prolonged ED stay especially those treated with NIV, assigned lower priority triage scores at ED presentation, and non-trauma patients.

## Background

Rising demand for emergency department (ED) services and the relative shortage of hospital beds are major contributors to ED crowding and result in protracted ED length of stay (LOS) [1]. Delayed admission to an intensive care unit (ICU) from the ED may occur due to hospital crowding and a lack of available ICU beds [1,2]. ED crowding due to a lack of hospital beds is a global problem [3]. In the US, the proportion of critically ill patients presenting to EDs is rising and their ED LOS is increasing [4-6].

Management of critical illness is highly resource intensive and time-sensitive [7]. Among patients who present to an ED, critically ill ventilated patients require a high level of care and are at high risk of adverse events. Expedited admission (under 2 h) from the ED to the ICU of critically ill ventilated patients has been associated with shorter durations of mechanical ventilation and ICU LOS [8]. Insufficient monitoring and/or substandard management of these patients may pose a substantial threat to patient safety, leading to complications and adverse outcomes. Key quality of care measures may be difficult to implement in the busy ED setting. Examples of these measures include patient repositioning to reduce atelectasis and semi-recumbent positioning and provision of oral hygiene to prevent ventilator associated pneumonia [9]. Most EDs do not have resources for longitudinal critical care delivery such as uninterrupted 1:1 nurse-to-patient ratios [10], and availability of respiratory therapists (RTs), subspecialty expertise, and invasive hemodynamic monitoring.

Our objective was to quantify the ED LOS for patients requiring invasive and non-invasive ventilation (NIV) in four EDs and to explore patient and system level predictors of prolonged ED LOS. Additionally, we aimed to describe practices of delivery and monitoring of ventilation in the ED.

## Methods

### Study design and setting

We conducted a six-month prospective observational study of mechanical (invasive and NIV) ventilation utilization at four metropolitan EDs in Toronto, Canada. Two EDs were located in large community teaching hospitals and each had an annual census of over 65,000 patients. The other two EDs were in university-affiliated hospitals with regional trauma center designation and had an annual census of over 40,000 patients each. The interprofessional team of the four EDs included RTs, allied health professionals trained in the management of ventilation, who attended intubation and initiation of ventilation and were available on call if needed. The study was approved by the Research Ethics Boards (REB) of all participating hospitals (Saint Michael’s Hospital, Sunnybrook Health Sciences Centre, Toronto East General Hospital and Saint Joseph’s Hospital) and the University of Toronto who together waived the requirement for consent due to the observational nature of the study. All data were collected in an anonymized fashion and stored in a locked research office and on a secure computer server.

### Study population

We collected data on consecutive adult patients aged 16 and over who received invasive (endotracheal tube or tracheostomy) and/or NIV (administered by mask) mechanical ventilation. For feasibility reasons such as timing of REB approvals and availability of research personnel, data were collected from January to July 2009 at two sites and August 2009 to January 2010 at the remaining two EDs. For patients with multiple ED presentations, we included data from the first ED presentation only.

### Data collection

Mechanically ventilated patients were identified by the treating respiratory therapists (RTs) during the ED visit. RTs notified the study team of patient enrolment by telephone and completed a short data collection form identifying the primary reason for ventilation, time of intubation and initiation of mechanical ventilation (invasive or non-invasive), type of monitoring used, reasons for delayed discharge, and disposition location. Admission logs of hospital departments likely to receive ventilated patients from the ED were screened to identify missed patients.

We collected demographic data, Canadian Triage Acuity Scale (CTAS) score assigned by the triage nurse on ED presentation, location of intubation, requirements for sedation and vasopressors, worst (furthest from normal) systolic blood pressure, heart rate and oxygenation saturation during ED stay, ED time indicators (triage, registration, departure readiness, and departure), and ED discharge diagnoses. ED discharge delay was determined when the difference between ED departure readiness and ED departure exceeded 6 h. A CTAS score of 1 represents a patient requiring immediate assessment and intervention, CTAS 2 patients have a maximal wait time for assessment by a physician of 15 min, and patients triaged as CTAS 3 require assessment within 30 min [11]. ED discharge diagnoses were later classified according to the Canadian Emergency Department Diagnoses Shortlist [12].

We followed patients to determine the duration of mechanical ventilation, ICU (where applicable) and hospital LOS, and vital status at discharge. The duration of ventilation was defined as the time from initiation in a participating ED until either successful extubation, disconnection from positive pressure ventilation for tracheostomized patients, death, or transfer to another institution. We also collected initial ventilator settings, any ventilator adjustments performed in ED, and arterial blood gas (ABG) values before and after mechanical ventilation initiation. For patients receiving NIV, the number of times NIV was discontinued and reinitiated and the reason for discontinuation were recorded. We summed the duration of each NIV episode to obtain the total duration of NIV in the ED. ED demographic data and six-month summary activity statistics, obtained from ED directors and ED information systems respectively, were also captured.

### Data analysis

We summarized continuous variables using means and standard deviations or medians and interquartile ranges depending on the data distribution and categorical variables using proportions and their 95% confidence intervals (CI). We calculated the ED LOS using time-to-event methods (survival analysis), with censoring of deaths, for (1) all patients, and (2) excluding trauma diagnoses. We compared differences between groups using the log-rank test. We constructed a Cox proportional hazard model to estimate the effect of selected covariates on the time to ED discharge for non-trauma patients receiving only invasive ventilation. We excluded trauma patients from our model due to the presence of trauma activation protocols designed to reduce ED LOS in the two designated trauma centres. The Cox model included covariates representing patient demographics and a priori selected variables deemed likely to be associated with ED LOS or showing bivariate associations (p < 0.10) and adjusted for the correlation among patients in the same hospital. We calculated the relative risk (RR) of an ED ‘discharge delay’, defined as the time between discharge readiness and physical departure, of > 24 h for invasively ventilated patients compared to those receiving NIV and both forms of ventilation. Variables likely to be associated with ABG measurement in the ED (binary of yes/no) after commencement of ventilation (ED LOS, ventilation type, and primary reason for ventilation) were selected a priori and examined using multiple logistic regression. The model was assessed for collinearity and goodness of fit [13]. All tests were two-tailed and we considered a p-value of 0.05 as statistically significant. Analyses were performed using SPSS 18.0 (SPSS, Chicago, IL, USA) and SAS 9.1 (SAS Institute, Cary, NC, USA).

## Results

### Patient characteristics

We recorded data on 618 patients receiving ventilation in the ED representing 631 ED presentations; 484 (78.3%) patients received invasive ventilation only, 118 (19.1%) NIV; 16 (2.6%) patients received both. These 618 patients represented 0.5% (95% CI 0.4–0.5) of the 135,352 patients seen at participating EDs during the study period; 353/1,828 (19.3% [95% CI 17.5–21.1]) CTAS 1 patients, 237/25,724 (0.9%, [0.8–1.0]) CTAS 2, and 26/71,502 (0.04% [0.03–0.05]) CTAS 3 patients (2/618, 0.3% not reported).

We present patient demographics according to type of ventilation in Table 1. Of the 367 patients presenting to EDs with trauma center designation, head injury (118, 32.2% [27.4–36.9]), trauma without head injury (40, 10.9% [7.7–14.1]), and primary neurologic disorder (67, 18.3% [14.3–22.2]) were the most frequent indications for invasive ventilation. In the two community EDs, cardiac arrest (30/117, 25.6% [17.7–33.6]), overdose (26, 22.2% [14.7–29.8]), and acute respiratory failure (14, 12.0% [6.1–17.9]) were the most frequent indications.

**Table 1:** Patient Characteristics

	**MV only (n = 484)**	**NIV only (n = 118)**	**Both (n = 16)**
Age, *median (IQR)*	58 (41–76)	76 (64–84)	72 (62–81)
Male gender	307 (63.4)	64 (54.2)	8 (50.0)
CTAS codes^a^			
1	326 (67.4)	22 (18.6)	5 (31.3)
2	139 (28.7)	87 (73.7)	11 (68.8)
3	19 (3.9)	7 (5.9)	–
Not reported	–	2 (1.7)	–
Reason for mechanical ventilation			
Trauma with head injury	123 (25.4)	2 (1.7)	–
Primary neurologic disorder	80 (16.5)	2 (1.7)	1 (6.3)
Cardiac/respiratory arrest	61 (12.6)	–	1 (6.3)
Trauma (not including head)	43 (8.9)	–	–
Overdose	40 (8.3)	1 (0.8)	–
Pneumonia	34 (7.0)	12 (10.2)	2 (12.5)
Acute respiratory failure^b^	34 (7.0)	19 (16.1)	1 (6.3)
Heart failure	12 (2.5)	45 (38.1)	4 (25.0)
Sepsis and septic shock	29 (6.0)	–	2 (12.5)
COPD exacerbation	7 (1.4)	35 (29.7)	4 (25.0)
NMD	4 (0.8)	–	1 (6.3)
Asthma	1 (0.2)	2 (1.7)	–
Other	16 (3.3)	–	–

All values are n (%) unless indicated.

CTAS = Canadian triage and acuity scale, COPD = chronic obstructive pulmonary disorder, NMD = neuromuscular disease, MV = mechanical ventilation (invasive), NIV = non-invasive ventilation.

^a^CTAS score of 1 represents a patient requiring immediate assessment and intervention, CTAS 2 patients have a maximal wait time for assessment by a physician of 15 min, and patients triaged as CTAS 3 require assessment within 30 min. [11].

^b^Excludes patients with pneumonia, COPD exacerbation and asthma.

### ED length of stay

**Figure 1:**
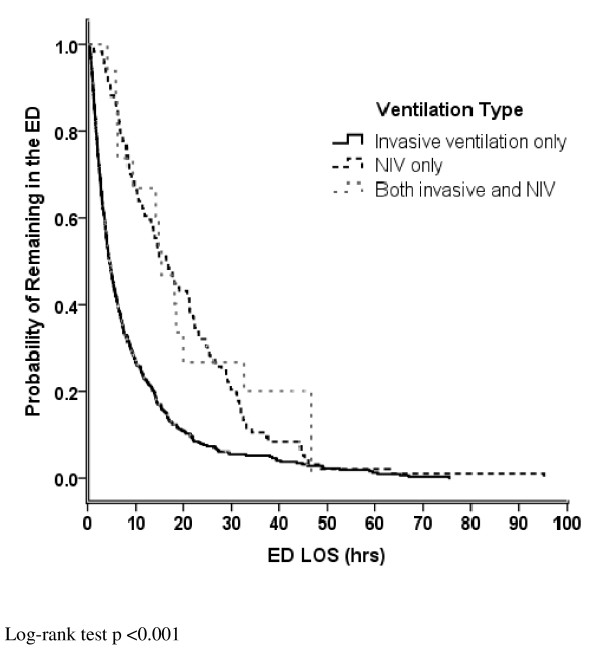
**Kaplan-Meier Estimated Probability of Remaining in the ED**. Legend:___ = invasive ventilation only ---- = non-invasive ventilation only, ---- = both invasive and non-invasive ventilation.

Kaplan-Meier estimated median (IQR) duration of ED stay for all patients was 6.4 h (2.8–14.6). Of the 618 patients, 237 (38.4% [34.5–42.2]) had a total ED LOS longer than 6 h but less than 24 h and 71 (11.5% [9.0–11.0]) had a total ED LOS longer than 24 h. Patients presenting to an ED requiring NIV had a longer median (IQR) duration of ED stay; 16.6 h (8.2–27.8) compared to 4.6 (2.2–5.3) hours for those receiving invasive mechanical ventilation exclusively and 15.4 h (6.4–32.6) for patients receiving both (*p* <0.001) (Figure 1). Median ED LOS differed according to site (range 3.7 to 14.4 h for invasively ventilated patients [*p* <0.001] and 11.4 to 29.8 h for NIV patients [*p* <0.001]). Patients with trauma diagnoses had a shorter median (IQR) LOS, 2.5 h (1.3–5.1), compared to ventilated patients with non-trauma diagnoses, 8.5 h (3.3–14.0) (*p* <0.001). Patients initially triaged as CTAS 3 were more likely to remain longer in the ED whereas those intubated at another ED prior to transfer had a shorter ED LOS after adjusting for age, gender and reason for ventilation (Table 2).

**Table 2:** Factors affecting ED LOS for Invasively Ventilated Non-Trauma Patients

	**Discharge**	**ED Alive**	**Univariate**	**Multivariate**
	**n/N**	**% [95% CI]**	**HR [95% CI]**	**HR [95% CI]**
Age	–	–	1.0 [1.0–1.0]	1.0 [1.0–1.0]
Male gender	176/192	92 [87–95]	1.0 [0.8–1.2]	1.0 [0.8–1.2]
Triage code				
CTAS 1	158/177	89 [84–93]	1	1
CTAS 2	116/121	96 [91–98]	0.8 [0.6–1.1]	0.8 [0.6–1.3]
CTAS 3	17/19	89 [69–97]	0.4 [0.3–0.6]	0.3 [0.2–0.4]
Location of intubation				
Study ED	215/228	94 [90–97]	1	1
Other ED	25/25	100 [−]	6.1 [3.0–12.3]	8.4 [5.4–12.9]
EMS	43/56	77 [64–86]	1.3 [0.8–2.1]	1.5 [1.0–2.4]
Reason for ventilation				
Respiratory/cardiac	112/117	96 [90–98]	1	1
Neurological	116/120	97 [92–99]	1.2 [0.8–1.7]	0.9 [0.7–1.2]
Cardiac arrest	44/60	73 [61–83]	0.8 [0.4–1.6]	0.9 [0.5–1.8]
Other	19/20	95 [76–99]	1.0 [0.7–1.4]	1.0 [0.8–1.2]

ED = emergency department, HR = hazard ratio, CI = confidence interval, CTAS = Canadian triage and acuity scale, EMS = emergency medical services

n/N refers to the number of patients with the identified characteristic (e.g. male gender) that were discharged alive from the ED out of the total number of patients in the study with the same characteristic.

A hazard ratio of >1 reflects a greater likelihood of discharge and hence a shorter length of stay.

‘Discharge delay’ > 6 h but ≤ 24 h was experienced by 97 (15.7% [12.8–18.6]) patients. Discharge delay > 24 h was experienced by 46 (7.4% [5.4–9.5]) patients. Patients receiving NIV or both forms of ventilation were more likely to experience a discharge delay > 24 h compared to patients receiving only invasive ventilation, RR 4.1 (95% CI 2.3–7.3) and RR 5.8 (95% CI 2.2–14.8), respectively. The primary reason for discharge delay for patients receiving invasive ventilation was lack of an available ICU bed (55 of 64 patients that had reason for discharge delay reported, 85.9% [75.4–92.4]). The primary reason for discharge delay for NIV patients was awaiting treatment decision (34/60, 56.7% [44.1–68.4]; lack of available in-patient bed was identified for 23/60 (38.3% [27.1–51.0] patients. Patients receiving invasive ventilation in the ED remained in hospital for a median (IQR) of 16.4 days (5.4–38.7), NIV 8.8 days (4.3–21.2) and those receiving both invasive and NIV 22.4 days (10.7–28.5) (p <0.001).

Hospital mortality was 36.1% (95% CI 32.2–39.9). ED and hospital discharge outcomes are shown in Table 3.

**Table 3:** Emergency Department and Hospital Discharge Destinations

**Discharge Destinations**	**MV only (n = 484)**	**NIV only (n = 118)**	**Both (n = 16)**
From the ED			
Intensive care unit	327 (67.6)	20 (17.0)	9 (56.3)
Operating room	45 (9.3)	–	–
Death	33 (8.8)	5 (4.2)	3 (18.8)
Other hospital	33 (6.8)	–	1 (6.3)
General floor^a^	17 (3.9)	67 (56.8)	2 (12.5)
Coronary care/high dependency unit	18 (3.5)	22 (18.6)	1 (6.3)
Home^a^	11 (2.3)	4 (3.4)	–
From the hospital	(n = 406)	(n = 109)	(n = 12)
Home	160 (39.4)	63 (57.8)	6 (50.0)
Death	146 (36.0)	29 (26.6)	5 (41.7)
Other acute care hospital	38 (9.4)	7 (6.4)	–
Rehabilitation	35 (8.6)	2 (1.8)	1 (8.3)
Long term care facility	21 (5.2)	4 (3.7)	–
Not available	6 (1.5)	4 (3.7)	–

MV = mechanical ventilation (invasive), NIV = non-invasive ventilation, ED = emergency department.

^a^Of the 27 patients discharge to the floor or home, the 3 main indications for ventilation were overdose (12, 44.4%), trauma (8, 29.6%) and cardiac arrest (3, 11.1%).

### Ventilation and monitoring

Pressure control modes were used most frequently 230/500 (46.0% [41.6–50.4]) during invasive mechanical ventilation, followed by Assist Control (volume) 180/500 (36.0% [31.8–40.2]) and spontaneous modes 58/500 (11.6% [8.8–14.4]) (missing for 32 patients). NIV was administered as noninvasive positive-pressure ventilation (NPPV) using an oro-nasal face mask for all patients. Twenty-seven (20.1% [13.4–26.9]) NIV patients refused invasive ventilation, of whom 13 (48.1% [29.3–67.0]) survived to admission but died in hospital. Twelve patients required a recommencement of NIV in the ED after initial discontinuation. Of the 16 patients receiving both types of ventilation, 14 received NIV prior to intubation constituting a NIV failure rate of 14/134 (10.5% [5.3–15.6]). Congestive heart failure and chronic obstructive pulmonary disease were the primary reasons for NIV in 8 (57.1% [32.6–78.6]) of these patients. The median (IQR) time from NIV initiation to intubation was 1.25 h (1.25–5.0). The remaining two patients received NIV after extubation in the ED.

ABG measurement following commencement of ventilation was performed on 341/618 (55.2%) patients. The median (IQR) time until availability of ABG results from commencement of invasive or NIV was 88 min (38–190). The likelihood of ABG measurement was influenced by site, ED LOS, neurological impairment for reasons other than trauma, and ventilation type (Table 4). Of the 277 patients without ABG assessment in the ED, 76 (27.4% [22.2–32.7]) had end-tidal CO_2_ monitoring, and all patients had oxygen saturation monitoring. For patients presenting to an ED with traumatic brain injury, 7/99 (7.1% [2.0–12.1]) had an initial PaCO_2_ ≤ 30 mmHg and 16/99 (16.2% [8.9–23.4]) > 49 mmHg.

**Table 4:** Arterial Blood Gas Analysis in the ED

	**No ABG**		**Univariate**	**Multivariate**
	**n/N**	**% [95% CI]**	**OR [95% CI]**	**OR [95%*CI***]
Site				
ED1	73/222	33 [27–39]	1	1
ED2	119/199	60 [53–67]	3.0 [2.0–4.5]	2.5 [1.6–3.9]
ED3	65/99	66 [56–75]	3.9 [2.4–6.4]	3.7 [2.1–6.7]
ED4	20/98	20 [12–28]	0.5 [0.3–0.9]	0.6 [0.3–1.2]
ED LOS (h)	–	–	1.0 [0.9–1.0]	1.0 [0.9–1.0]
Ventilation type				
Invasive	215/484	44 [40–49]	1	1
Non-invasive	54/118	46 [37–55]	1.1 [0.7–1.6]	1.6 [0.8–3.3]
Both	8/16	50 [26–75]	1.3 [0.5–3.4]	5.3 [1.5–19.2]
Reason for ventilation				
Respiratory/cardiac	70/182	39 [31–46]	1	1
Trauma	65/168	39 [31–46]	1.0 [0.7–1.6]	0.9 [0.6–1.6]
Neurological	66/124	53 [45–62]	1.8 [1.1–2.9]	1.9 [1.1–3.3]
Cardiac arrest	32/62	20 [14–26]	1.7 [1.0–3.1]	1.9 [1.0–3.9]
Other	12/21	57 [36–78]	2.1 [0.9–5.3]	2.4 [0.9–6.5]

ABG = arterial blood gas, OR = odds ratio, CI = confidence interval, ED = emergency department, LOS = length of stay.

## Discussion

Our results indicate that while ventilated patients represent a small proportion of ED visits, they are not a ‘rare’ event and remain in the ED for prolonged periods of time, given the resource intensive nature of their care. ED LOS for patients requiring NIV was substantially longer than for patients receiving invasive ventilation exclusively. During stabilization of NIV patients appropriate disposition may be difficult to determine in that it may be unclear if the patient requires admission to ICU or to a step-down unit or floor. This may contribute to prolonged ED LOS as admitting teams (ICU and non-ICU) may delay patient acceptance until the patient's response to NIV is ascertained. Additionally options for provision of NIV outside of ED and the ICU may be limited. In our study sites, one ED could not provide NIV in an alternative setting other than ICU; disposition options were limited to coronary care and step down units in the remaining EDs. Review of admission pathways for these patients and consideration for provision of NIV on general floors may be warranted.

A substantial number of patients experienced delayed departure from the ED after being deemed departure ready. Delays in hospital admission contribute to ED crowding and jeopardize the safety and quality of care delivered to both critically and non-critically ill patients. EDs often lack the resources and personnel trained in the longitudinal management of the critically ill [14-16]. Training of ED physicians and nurses in the ongoing management of ventilated patients is generally limited [17]. In North America, mechanical ventilation is initiated by RTs, who typically leave the ED once the patient has been stabilized and return for reassessment and to facilitate transfers, at the request of medical or nursing staff. Together, these factors may result in ventilated patients in the ED receiving suboptimal and interrupted care. Similarly, in countries that do not utilize RTs such as Europe, Scandinavia, the United Kingdom, and Australasia clinicians specialized in the management of ventilation may not be present in the ED at all times.

Protracted ED LOS is a marker of hospital access block and ED crowding and may increase hospital mortality for critically ill and non-critically ill patients [2,18-24]. Hospital LOS and mortality for our patient cohort were similar to those reported in a large international cohort study of patients receiving mechanical ventilation in the ICU [25] and comparable to the ICU mortality rate reported in a multi-center Canadian cohort study [26]. We did not specifically examine the relationship between hospital mortality and ED LOS due to the lack of validated and reliable tools to measure illness severity in the ED [27]. Future studies are required to develop and validate ED illness severity measures to enable characterization of the association between ED LOS and hospital mortality for critically ill patients.

In our study ED LOS for patients with non-trauma diagnoses differed according to site, CTAS score, and the location of intubation. Differences across sites likely reflect differences in ICU and hospital capacity and potentially modifiable bed management practices, while CTAS scores reflect differences in patient acuity. Detecting differences across sites may assist with identification of modifiable risk factors and best practices that can be transferred to other institutions. Patients transferred from an ED other than a study ED experienced shorter ED stays as presumably these were anticipated transfers for specialized services within tertiary care centers.

We noted a preference for pressure control ventilation modes for all patient indications. Clinicians may prefer for this ventilation style due to the decelerating flow pattern and ability to limit inspiratory pressures [28-30]. Ventilator induced lung injury may result from choice of ventilation modes or strategies that do not limit inspiratory pressures and deliver tidal volumes based on predicted body weight [31,32]. We were unable to calculate tidal volume by predicted or actual body weight as patient height and weight were rarely documented either on our case report forms or on documents used by participating EDs and ICUs. It is unclear if this indicates clinicians were not targeting tidal volume to predicted body weight or if it reflects a need to revise ventilation charting in the ED.

Recommendations for ABG sampling state ABGs should quantitate the response to therapeutic interventions such as mechanical ventilation [33]. Only 55% of patients had an ABG measured during their ED stay and the median time to availability of ABG results was greater than one hour. Point of care ABG analysis was unavailable at the participating EDs. Of concern was the number of traumatic brain injured patients with a PaCO_2_ less than 30 and greater than 49 mmHg on initial measurement. Studies indicate PaCO_2_ levels in this range result in increased mortality [34,35].

## Limitations

This study was conducted in four centers representing inner city, high volume EDs, two of which provided regionalized trauma services to the province of Ontario. Our findings may not be generalizable to other centers and countries that may vary in their ability to provide care to ventilated patients and experience differences in patient and staffing profiles, ED LOS, and access block. RTs were asked to identify the primary reason for mechanical ventilation at the time of initiation from a predefined list and reasons for ED discharge delay introducing the potential for subjective assessments. Notwithstanding, reasons for ventilation were checked against ED discharge diagnoses and occurrence of ED discharge delay was confirmed in ED information systems. Due to the observational nature of the study, other confounding factors may have influenced ED LOS that were not accounted for in multivariate analyses. As well we could not examine the relationship between hospital mortality and ED LOS in the absence of a reliable ED illness severity score.

## Conclusions

While patients requiring mechanical ventilation represent a small proportion of the total ED presentations they are not an infrequent event and many experienced an ED LOS > 24 h. This finding raises concerns about the potential impact on patient safety and the quality of care received by critically ill patients in the ED and suggests a need for education for the ED team on management of ventilation beyond initial stabilization. ED discharge delays occurred more frequently for patients receiving NIV in the ED suggesting the need for review of admission pathways for these patients and consideration for provision of NIV on general floors.

## Abbreviation

ABG: Arterial blood gas; CI: Confidence interval; CTAS: Canadian triage and acuity scale; ED: Emergency department; ICU: Intensive care unit; IQR: Interquartile range; LOS: Length of stay; NIV: Non-invasive ventilation; OR: Odds ratio; RR: Relative risk; RT: Respiratory therapist.

## Financial support

Canadian Association of Emergency Physicians and the Canadian Institutes of Health Research Partnerships in Health Service Improvement. Dr. Burns holds a Clinician Scientist award from the Canadian Institutes of Health Research.

## Competing interests

The authors have no potentially conflicting personal or financial interests to be declared.

## Author contributions

LR, SG, KB, AW, JL conceived the study, LR coordinated data collection and managed the data entry process, AK performed data analyses, all authors contributed to interpretation of the data, manuscript drafts and have read and approved the final manuscript.
